# New Investigations Concerning Epilepsy, Resulting from Certain Lesions of the Spinal Nerve

**Published:** 1869-06

**Authors:** 


### NEW INVESTIGATIONS CONCERNING EPILEPSY, RESULTING FROM CERTAIN LESIONS OF THE SPINAL NERVE.

BY DR. BROWN-SÉQUARD.

Epilepsy is an affection of man so terrible and so difficult to cure, that it is important to collect with the greatest care all facts capable of affording any light upon so frightful a malady.

In the year 1850, I found that certain lesions of the spinal cord would produce epileptiform convulsions among Guinea-pigs; and since that time I have not ceased to experiment upon the numerous epileptic animals which I have had constantly at my disposal. During the winter of 1866-7, after having submitted a great number of Guinea-pigs to the principal lesions which produce epilepsy, I had opportunity to make many new observations that have not been given to the public as yet, except very briefly, in my course at the Medical College of Cambridge University (United States), and in two recent communications to the Academy of Medicine, at Paris. I propose to present here, with some detail, the greater part of these, as well as some previous observations relative to the physiology of epilepsy:—

I. LESIONS WHICH PRODUCE EPILEPSY AMONG GUINEA-PIGS.

In a paper read at the Academy of Science, January 1st, 1856, I announced the following lesions of the medulla spinalis as capable of producing this affection:—

1st. Complete, or nearly complete, transverse section of one lateral half.

2d. Simultaneous transverse section of the posterior cords of the posterior gray cornua, and of one part of the lateral cords.

3d. Transverse section, either of the two posterior or the lateral cords, or, finally, of the anterior cords only.

4th. Complete transverse section.

5th. A simple puncture.

Without exception, all the Guinea-pigs that have survived the first, second, or fourth of these wounds more than five or six weeks have been attacked by epilepsy.

I can repeat, then, to-day, what I said in 1856, that a complete transverse section of the spinal nervous centre, or of a little more than the posterior half of this organ, or, finally, of one of its lateral halves, after a certain length of time, invariably produce a convulsive epileptiform affection among Guinea-pigs, which, as I shall show hereafter, has all the essential characteristics of epilepsy in the human being.

Of the three great white cords of the medulla spinalis—the anterior, the lateral, and the posterior—the section of the last, especially, is capable of producing epilepsy. But *complete* epilepsy does not usually follow injuries, limited to any one of these parts.

The section of one or the other of these cords, on one side only, rarely occasions complete epilepsy; the same is true of the simultaneous transverse section of one of the posterior gray cornua, and of some fibres of the two neighboring white cords.

Moreover, as I had already discovered in 1856, a fully developed form of epilepsy may be the result of a simple puncture of the spinal nerve, particularly in its posterior half.

Among Guinea-pigs that do not become epileptic after an injury of the spinal nerve, it is quite frequent, after irritating certain portions of the skin, to notice some reflex convulsive motion of the face, or the members not paralyzed. The agitation is similar to that observed among animals which should become epileptic a few days or a week before the appearance of a complete attack. It may be considered, therefore, of an epileptic nature.

II. PARTS OF THE NERVOUS SYSTEM, THE LESION OF WHICH FREQUENTLY OR ALWAYS PRODUCES EPILEPSY AMONG GUINEA-PIGS.

It is probable that nearly if not all the parts of the medulla spinalis, from the origin of the first pair of cervical nerves to the coccygeal termination of this nervous centre, are capable of producing epilepsy, after receiving a wound by incision. But the affection never fails to occur after an injury of the part, extending from the seventh or eighth dorsal vertebræ to the second or third lumbar, if the operation is one of those I have previously indicated as invariably followed by epilepsy. The spinal marrow, from the third lumbar vertebra to the coccygeal termination, loses continually its power to occasion this malady.

I am not prepared to say whether the part of the medulla spinalis which gives origin to the last two cervical and the first four dorsal pairs can produce epilepsy or not. When I have removed a complete or nearly complete lateral half of the nervous centre, in this region, the animals have all died before the time for the appearance of the paroxysm. After the section of the posterior half of the spinal cord, or of one of the lateral halves, between the second and fifth pairs of nerves in the cervical region, if death should not occur in less than four or five weeks, epilepsy usually manifests itself. It is evident from these facts that nearly all parts of the spinal nerve, considered in its length, can produce epilepsy when irritated by incision. But all these parts, except that having its centre in the region just indicated, possess, only partially, the power of engendering this disease. It can not be said that epilepsy always or frequently follows extensive lesions of the rachidien bulb among these animals. After such operations, the duration of life is far too short to render the solution of this question possible.

Meanwhile, I have frequently seen Guinea-pigs survive for many months a transverse section of the restiform body, of the intermediate cord, or of one of the anterior pyramids; and I have never seen among them either simple, reflex convulsions, in the parts not paralyzed, or epileptic attacks, spontaneous or provoked. At the present time, there are near me seven of these creatures, having the restiform body of one or both sides cut transversely in the vicinity of the calamus scriptorius. With the exception of anæsthesia in some parts, and a little hyperæsthesia in others, they are, to all appearance, in perfect health, with no paralysis of the body or limbs. There have been no spontaneous convulsions among them, and the liveliest irritations of the skin in the region of the head, neck, etc., have never occasioned spasmodic phenomena. Some were submitted to the operation six months ago, others only two months since.

Guinea-pigs have frequently lived after I have removed the V of gray substance of the calamus scriptorious, and a small quantity of the neighboring gray substance. Convulsions have never followed this operation, save in one instance. But in this exceptional case, the attacks of epilepsy were the most violent I have ever witnessed among these creatures. From this animal I removed the part of the rachidien bulb, which Flourens calls the centrum vitale. The operation was made during one of my lectures at the Royal College of Surgeons, in London, May, 1858. The subject died from accident in September, 1860, having had, meanwhile, at least five or six convulsions daily for several months preceding its death. I shall have occasion to say hereafter that its attacks differed in many respects from those caused by wounds in the lumbar or dorsal regions.

Injury of the nerves may also give rise to epilepsy among

Guinea-pigs. Twice, I have observed this disorder after section of the sciatic nerve,* and I have frequently seen it follow section of the roots of four or five dorsal nerves on one side. Have I wounded the spinal cord in making the section of the roots? It seems to me absolutely impossible that it should be thus in every case, and, therefore, I believe it safe to conclude that epilepsy can be produced among these creatures, by sections of the roots of the spinal nerves.

The facts I have just mentioned are quite valuable for the demonstration of a relation of causality between the injuries of the medulla spinalis and of other parts of the nervous system, on one side, by the frequent appearance of epilepsy, and on the other, by the non-existence of spontaneous epilepsy, or at least its extreme rarity in this species of animals, in which these operations produce more or less frequently this nervous affection. For 30 years, I have had near me a considerable number of Guinea-pigs (certainly many thousands), and I have never observed epileptic attacks among them, except in those submitted to the operations specified, or among their descendants. That cannot authorize me to affirm that idiopathic epilepsy never exists in this species of mammifera, but at least it suffices to show that epilepsy if the frequent attendant of certain lesions, is, assuredly, the result of these lesions.

III. TIME OF THE APPEARANCE OF EPILEPSY AFTER INJURIES OF THE SPINAL NERVE AMONG GUINEA-PIGS.

I have said, previously, that this convulsive affection manifests itself in the third week after the operation. This is true, ordinarily, of the simple reflex convulsions; but complete epilepsy usually appears only in the fourth or fifth week after the injury. Sometimes the convulsions manifest themselves much sooner—in one case, at the end of six days, and in some others, in eight or ten days. Without exception, all animals that have

*My colleague, M. Vulpian, told me that he had observed epilepsy in a Guinea-pig possessing the sciatic nerve. Aside from this case, he has never seen the disease among these animals, from which we may conclude that it must be extremely rare, when we consider the immense number of Guinea-pigs that he has near him, in the laboratory of Flourens, and elsewhere.

survived for a sufficient time one of the lesions invariably followed by epilepsy have had their first attack previous to the end of the eighth week.

Animals well fed, and surrounded by good hygienic influences, are attacked much later than those poorly fed, exposed to cold, dampness, etc.

The more extensive the injury of the spinal nerve the sooner in general epilepsy ensues.

Among very young Guinea-pigs this affection is usually more tardy in making its appearance than among those three or four months old.

IV. PARTS OF THE SKIN WHICH ARE CAPABLE, WHEN IRRITATED, OF PRODUCING AN ATTACK OF EPILEPSY AMONG GUINEA-PIGS, HAVING UNDERGONE AN OPERATION OF THE SPINAL NERVE.

For some time, I believed that after a section of the lateral half of the nerve, in the vicinity of the tenth dorsal vertebra, epileptic convulsions were provoked by irritation of the hyperæsthetic member. The supposition was quite natural, as there was no reason to foresee or even to suppose that, in a region remote and anterior to the place of injury to the vertebral column, any particular part of the skin when irritated was alone capable of producing such results. In order to provoke the attack, I pinched the hyperæsthetic member, while holding the animal in my left hand with one or two fingers touching the face and neck. There was immediate distress and very great agitation of the animal, producing some friction of the face and neck, that is, an irritation of the only parts really capable of causing the paroxysm. When this took place, under such conditions, it seemed apparent that the irritation of the hyperæsthetic member had engendered it. But after finding the convulsions also occurred, when I held, in the same manner, a Guinea-pig that had undergone a complete transverse section of the dorsal nerve, and, consequently, no sensibility existing in the posterior region, the irritation of which only produced reflex motion, it became evident that my first opinion was false. I was then led to inquire what were the parts of the anterior region the irritation of which would cause the attack? and I

was not long in deciding that the face was one of these parts. I immediately extended my investigations in this subject, and I ascertained:—1st. That it is only on the side of a unilateral lesion of the spinal nerve that certain portions of the skin acquire the faculty of occasioning an attack of epilepsy when irritated. 2d. That these portions of the skin are those of one part of the face and neck. 3d. That the nerves which are distributed to these portions of the skin originate from the trigeminal and the second and third pairs of rachidian nerves.

My latest investigations in this subject confirm what I had already briefly announced in 1856, at the Academy of Medicine, that the irritation of a certain zone of the skin would produce epileptic paroxysms, in cases where epilepsy had followed the section of one lateral half of the spinal cord. The limits of this zone, as I have discovered them, are represented by the dotted lines in Figure 1.

These limits, as it appears, are slightly curved lines which circumscribe an ovular space, which, in the adult Guinea-pigs, is about five centimetres long, and three and a-half or three and three-fourths centimetres in width. If we should draw, as in the figure just mentioned, a line from the anterior palpebral-angle to the projection of the superior maxillary bone, which forms the lower limit of the suborbitar fossa, from there to the middle of the lower jaw, from this point, passing below the angle of the jaw to the scapulo humeral articulation, thence reascending along the anterior edge of the shoulder-blade, until the middle point of its length is reached, from this last point into the attachment of the lobe of the ear, and, finally, from there to the point of departure, the anterior palpebral angle, passing below and very near the edge of the lower eyelid, we inclose the excitable region. All other parts of the body, including the hyperæsthetic portions of the skin, back of the seat of hemisection of the spinal nervous centre, without producing any spasmodic action, without even any convulsive movements of the face, head, and eyes, which are frequently manifested after very slight irritation of the zone capable of producing an attack.

On the head and neck, we can, without exciting convulsions, irritate severely the skin or membrane of the nose, lips, tongue, conjunctiva, upper eyelid, edge of the lower eyelid, space comprized between the eyes, ears, shoulders, and head, and also between the lower jaws (except in a very short space along the inferior maxillary). I repeat, then, there is only the zone of skin on the head and neck, that may be capable under the influence of severe or trivial irritation of occasioning epileptic spasms, among Guinea-pigs that have been submitted to the operation designated.

It is not always thus in cases where there has been a complete or nearly complete transverse section of the spinal cord. On the contrary, it is most common to find that besides the zone described above, and which in these cases, on both sides, has power to cause convulsions, other parts of the skin possess the same faculty. The skin between the right and left zones, which extends over the last four cervical vertebra, and, especially, that portion comprised between the scapulæ, has also power to cause attacks of complete epilepsy, or spasmodic action of some muscles of the face, neck, and anterior members. This zone, intermediate to the other two, sometimes extends back even to the region of the cicatrix, where the incision was made, and the vertebral canal was opened to allow the section of the nerve. Sometimes I have even seen a paroxysm occur after irritation of the cicatrix; but, as in the very same animals where I have observed this phenomena, I have not been able to reproduce it in numerous experiments, it is possible that the local disturbance may have affected the parts of the skin anterior to the cicatrix, and thus have caused the attack by irritation of those portions possessing undue excitability. When the lesion, consisting of a transverse section of the posterior half of the spinal nerve, on both sides, is more extensive on one side than the other, it is found, very frequently, that only one side of the face or neck is capable of producing convulsions. In these cases, the zone of the skin which acquires this morbid property is always found on the side where the nerve has been more severely wounded. Then, sometimes, portions of the skin beyond the zone limited to one side of the neck and face, and pertaining to the intermediate zone just alluded to, have also the power to bring on the spasms. The portion comprised between the scapulæ especially possesses this faculty.

Now and then, too, a lesion of the two posterior thirds of the spinal cord, on both sides, renders both sides of the face and neck capable of producing the convulsions, the same as occurs after a complete transverse section of this nervous centre. But there is this difference in the two cases, that in the first, the intermediate zone of which I have spoken, does not acquire, usually, the power of causing an attack, while, in the second case, on the contrary, this almost invariably takes place.

In the zone of skin on the face and neck, the points seeming most excitable are found at the angle of the jaw, below the eye, and at the middle of the lower edge of the scapula.

V. NERVES WHICH ARE DISTRIBUTED IN THE ZONE OF SKIN CAPABLE OF CAUSING AN EPILEPTIC ATTACK.

Among Guinea-pigs having undergone a transverse section of one lateral half of the spinal cord, this sensitive zone of skin receives ramifications from one cranial nerve, the trigeminal, and some of the cervical pairs. Of the three great branches of the trigeminal, the ophthalmic does not furnish ramifications to this zone; the other two supply it, particularly by the infra-orbital and the auricular temporal. The posterior branches of the second, third, and fourth pairs of cervical nerves are especially distributed in this space.

As I have already said, when there is a complete or nearly complete transverse section of the spinal cord, beyond the ordinary zone, on both sides of the head and neck, there is an intermediate zone over the last cervical vertibræ, and a part of the dorsal vertibræ, also capable of causing spasms when irritated. The branches of nerves which distribute themselves in this new zone of skin originate nearly, if not entirely, from the posterior branches of the spinal nerves, in a part of the cervical and dorsal regions.

(*To be continued.*)

